# Induced protection from a CCHFV-M DNA vaccine requires CD8^+^ T cells

**DOI:** 10.1016/j.virusres.2023.199173

**Published:** 2023-07-24

**Authors:** Joseph W. Golden, Collin J. Fitzpatrick, John J. Suschak, Tamara L. Clements, Keersten M. Ricks, Mariano Sanchez-Lockhart, Aura R. Garrison

**Affiliations:** a Virology Division, United States Army Medical Research Institute of Infectious Diseases, Fort Detrick, MD 21702, United States; b Center for Genome Sciences, Molecular Biology Division, United States Army Medical Research Institute of Infectious Diseases, Fort Detrick, MD 21702, United States; c Diagnostic Systems Division, United States Army Medical Research Institute of Infectious Diseases, Fort Detrick, MD 21702, United States

**Keywords:** Crimean-Congo hemorrhagic fever, Correlates of protection, CD8+ T cells, DNA vaccine, Glycoproteins, M-segment

## Abstract

Crimean-Congo hemorrhagic fever (CCHF) is a World Health Organization prioritized disease because its broad distribution and severity of disease make it a global health threat. Despite advancements in preclinical vaccine development for CCHF virus (CCHFV), including multiple platforms targeting multiple antigens, a clear definition of the adaptive immune correlates of protection is lacking. Levels of neutralizing antibodies in vaccinated animal models do not necessarily correlate with protection, suggesting that cellular immunity, such as CD8^+^ T cells, might have an important role in protection in this model. Using a well-established IFN-I antibody blockade mouse model (IS) and a DNA-based vaccine encoding the CCHFV M-segment glycoprotein precursor, we investigated the role of humoral and T cell immunity in vaccine-mediated protection in mice genetically devoid of these immune compartments. We found that in the absence of the B-cell compartment (µMT knockout mice), protection provided by the vaccine was not reduced. In contrast, in the absence of CD8^+^ T cells (CD8^+^ knockout mice) the vaccine-mediated protection was significantly diminished. Importantly, humoral responses to the vaccine in CD8^+^ T-cell knockout mice were equivalent to wild-type mice. These findings indicated that CD8^+^ T-cell responses are necessary and sufficient to promote protection in mice vaccinated with the M-segment DNA vaccine. Identifying a crucial role of the cellular immunity to protect against CCHFV should help guide the development of CCHFV-targeting vaccines.

## Highlights

• An M-segment based DNA vaccine, CCHFV-M_Afg09,_ protects mice from lethal challenge.
• CD8^+^ T-cell responses are necessary and sufficient for protection in CCHFV-M_Afg09_ vaccinated mice.
• The humoral response is dispensable for the protection provided by the CCHFV-M_Afg09_ vaccine.

## 1 Introduction

Crimean-Congo hemorrhagic fever virus (CCHFV) is the most widely distributed tick-borne virus of medical importance, with outbreaks occurring in the Middle East, Asia, Africa, and parts of Western Europe (reviewed in Bente et al. (2013)). CCHFV is sustained in an enzootic cycle by ticks and several vertebrate animals with humans as “dead-end” hosts. Even when sporadic, CCHF outbreaks have resulted in a global fatality rate of approximately 70%. Human infection frequently results from the bite of infected ticks, especially *Hyalomma* species (reviewed in Bente et al. (2013)). Contact with blood or tissues from infected livestock can also be a source of outbreaks in farming communities (Bakir et al., 2005; Leblebicioglu et al., 2015). Moreover, multiple cases of nosocomial infections have been reported because CCHFV spreads easily by human-to-human contact (reviewed in Bente et al. (2013), Vorou et al. (2007)). Presently, there are no internationally licensed vaccines, and treatment is limited to supportive care (reviewed in Bente et al., 2013). Since 2015, CCHF has been designated as a high priority emerging infectious disease by the World Health Organization (2015, 2018). This classification has led to an increased focus on the development of a CCHFV vaccine.

Classified within the *Orthonairovirus* genus of the *Nairoviridae* family, CCHFV has a tripartite, negative sense RNA genome comprised of a small (S), medium (M), and large (L) segment. The S segment encodes the nucleocapsid protein (NP) and a non-structural protein (NSs) by the positive-sense, the M-segment encodes the glycoprotein precursor complex (GPC), containing two glycoproteins (G_N_ and G_C_) as well as several non-structural proteins (mucin-like domain, GP38, GP160, GP85, and NS_M_), and the L segment encodes the RNA-dependent RNA polymerase (Schmaljohn and Nichol, 2007). To date, some vaccines targeting the CCHFV NP and/or the glycoproteins have been shown to be efficacious in animal models (Aligholipour Farzani et al., 2019a; Buttigieg et al., 2014; Suschak et al., 2021; Zivcec et al., 2018). Vaccine systems used to target CCHFV have included classical methods such as formalin or chloroform inactivated virus (Canakoglu et al., 2015; Papa et al., 2010), and more recently included genetic vaccine-based platforms, viral vectors, and virus-like replicon particles (VRPs) (Aligholipour Farzani et al., 2019b; Garrison et al., 2017; Hawman et al., 2021; Hinkula et al., 2017; Leventhal et al., 2022; Scholte et al., 2019; Suschak et al., 2021). While these vaccines have been effective in rodent (Aligholipour Farzani et al., 2019a; Buttigieg et al., 2014; Canakoglu et al., 2015; Garrison et al., 2017; Leventhal et al., 2022; Suschak et al., 2021) and nonhuman primate (NHP) systems (Hawman et al., 2021; Leventhal et al., 2022), the immune correlates critical for protection are not fully defined (Buttigieg et al., 2014; Garrison et al., 2017; Hawman et al., 2022; Kortekaas et al., 2015; Leventhal et al., 2022).

Our group developed a DNA vaccine expressing the full-length, codon-optimized M-segment, which encodes the structural and non-structural glycoproteins of the clinically relevant CCHFV-Afg09-2990 strain of CCHFV (Suschak et al., 2021). This vaccine (CCHFV-M_Afg09_) is highly immunogenic, eliciting both antigen-specific humoral and cellular immunity when delivered by intramuscular (IM) electroporation (EP). CCHFV-M_Afg09_ is completely protective against CCHFV-Afg09-2990 challenge in mice (Suschak et al., 2015). CCHFV only causes disease in rodents when type I interferon (IFN-I) is disrupted (Bente et al., 2010; Bereczky et al., 2010; Zivcec et al., 2013). Previously, we reported on an IFN-I antibody blockade model (IS) to study CCHFV infection in transgenic mice by transiently blocking this pathway. This was accomplished utilizing a commercially available murine non-cell depleting monoclonal antibody (mAb) targeting the IFNAR-1 subunit of the mouse IFN-α/β receptor (MAb-5A3) (Durie et al., 2022; Garrison et al., 2017; Golden et al., 2019; Golden et al., 2022; Lindquist et al., 2018; Sheehan et al., 2006; Smith et al., 2017). Disease in this model system is identical to that found in IFN-I knockout animals and has hallmarks of human disease, including liver injury. In addition, we have previously shown that the humoral immune response and protective efficacy of a CCHFV-M based DNA vaccine in both the commonly used IFN-α/β receptor knockout mouse (IFNAR^−/−^) and the IS model were comparable, with no significant difference in the total antibody response or the neutralizing antibody response (Garrison et al., 2017). Although we did not see an appreciable difference between the models with regards to the DNA vaccine, this model has an added advantage in that vaccine-mediated immune responses are produced in an IFN-I intact system as this pathway is only disrupted at the time of viral challenge. This is important because IFN-I is a central player in immune responses and a key contributor to effective antiviral responses (Bente et al., 2010; Bereczky et al., 2010; Braun et al., 2002; Gallucci et al., 1999; Luft et al., 1998; Zivcec et al., 2013). IFN-I triggers general antiviral states in cells and specifically regulates adaptive immune responses, triggering the expression of interferon-stimulated genes (ISGs). Ultimately, IFN-I modulates the effector function of immune cells (e.g., dendritic, B, and T cells) prompting the resolution of the infection. Here, we used this murine system to dissect the contribution of humoral and T-cell responses in M-segment targeting DNA vaccine protection against CCHFV. Utilizing different transgenic knockout (KO) mice, we address the specific contribution of the humoral (B cells) and cellular (TCR α/β T cells or only CD8^+^ T cells) immunity in the observed vaccine protection in this model.

## 2 Material and methods

### 2.1 Ethics statement

All animal research was conducted under a USAMRIID IACUC supported and approved protocol in compliance with the Animal Welfare Act, PHS Policy, and other Federal statutes and regulations relating to animals and experiments involving animals. The facility where this research was conducted is accredited by the Association for Assessment and Accreditation of Laboratory Animal Care International and adheres to principles stated in the Guide for the Care and Use of Laboratory Animals, National Research (Council, 2011). Humane endpoints were used during these studies, and mice that were moribund, according to an endpoint score sheet, were humanely euthanized. Mice were euthanized by CO_2_ exposure using compressed CO_2_ gas followed by cervical dislocation. However, even with multiple observations per day, some animals died as a direct result of the infection.

### 2.2 Mice

B6.129S2-*Tcra^tm1Mom^*/J (TCR KO), *B6.129S2- Ighm^tm1Cgn^*/J (B-cell KO), B6.129S2-*Cd8a^tm1Mak^*/J (CD8 KO), and C57BL/6 (B6), aged 6-8 weeks, were obtained from The Jackson Laboratory.

### 2.3 Virus

CCHFV strain Afg09-2990 was derived from a fatal human case in Afghanistan in 2009. Afg09-2990 was passaged three times in Vero cells (Bernhard Nocht Institute) and then propagated twice in Huh-7 cells at USAMRIID (Conger et al., 2015, Golden et al., 2019). Harvested virus was collected from clarified cell culture supernatants and stored at -80 °C. All CCHFV work was performed in BSL-4 containment.

### 2.4 DNA vaccination and viral challenge in mice

Groups of indicated strains of female mice (n=10-15) were vaccinated three times at 3-week intervals with 50 µg of the pWRG7077 DNA vaccine plasmid expressing the codon optimized Afg09-2990 M-segment open reading frame (CCHFV-M_Afg09_) by intramuscular electroporation (IM-EP) as previously described (Garrison et al., 2017). Control groups of 10-15 C57BL/6 mice were vaccinated concurrently by IM-EP with pWRG7077 empty vector. For IM-EP delivery, mice were anesthetized and then vaccinated in the tibialis anterior muscle with 20 μL of DNA solution using a 3/10 mm U-100 insulin syringe inserted into the center of an Ichor Medical Systems TriGrid electrode array with 2.5 mm electrode spacing. Injection of DNA was followed immediately by electrical stimulation at an amplitude of 250 V/cm, and the total duration was 40 ms over a 400 ms interval. Sera were collected prior to vaccination on days 0 and 42 by submandibular bleed. For the first experiment, a cohort of 5 mice per group were euthanized on day 49 for T-cell analysis. The remainder of mice were observed until day 63, when sera was harvested for antibody analysis. Mice were subsequently challenged on day 72. For challenge, all mice were treated by the intraperitoneal (IP) route with mAb-5A3 (Leinco Technologies Inc.) 24 h prior to (2.0 mg) and 24 h after (0.5 mg) CCHFV challenge. IS mice were challenged with 100 plaque forming units (PFU) of CCHFV strain Afg09-2990 by the IP route. The mice were monitored for 25 days for individual weight changes, clinical score, and survival.

### 2.5 T-cell ELISpot

Mouse T-cell ELISpot reagents were obtained from Mabtech. Antigen specific IFN-γ^+^ and IL-2^+^ T cells were quantified per manufacturer's instructions. Positive control wells were stimulated with 10 ng/mL PMA (Sigma-Aldrich) and 500 ng/mL ionomycin (Sigma-Aldrich). Test splenocyte wells were stimulated with the appropriate peptides at a concentration of 2.5 µg/mL as previously described (Suschak et al., 2021). Cells were incubated for 20 h at 37 °C in 5% CO_2_. Positive spots were visualized on a CTL Imager and counting was performed with Immunospot software (Cellular Technology Ltd.). Splenocytes from vaccinated mice were stimulated with pooled 15-mer peptides (2 pools of 17 peptides) containing a 5-base overlap in previously identified T-cell dominant regions of the Afg09-2990 M-segment open reading frames (Mimotopes).

### 2.6 MAGPIX antibody detection

Recombinantly expressed antigens from CCHFV (G_N_, NP, G_C_ and GP38) were purchased from Native Antigen Company. Magnetic microspheres and xMAP® antibody coupling kits were purchased from Luminex Inc. Phosphate buffered saline, Tween-20, and skim milk powder were purchased from Sigma-Aldrich. Goat α-mouse IgG and IgM (H&L) phycoerythrin conjugates were purchased from Thermo Fisher Scientific.

Antigens were covalently linked to microspheres following manufacturer's instructions. Briefly, 12.5 million microspheres were washed three times with 500 µL of activation buffer and resuspended in 274.5 µL of activation buffer. Next, 144.0 µL of sulfo-N-hydroxysulfosuccinimide and 81.5 µL of 1-ethyl-3-(3-dimethylaminopropyl) carbodiimide hydrochloride solutions were added, and tubes were gently rotated for 20 min. After activation, microspheres were washed three times with coupling buffer and antigen was added at 4 µg per million microspheres. The reaction was allowed to incubate for 2 h, after which, the microspheres were washed three times with 500 µL of PBS-T (phosphate buffered saline with 0.05% Tween-20), resuspended at 12.5 million microspheres per mL in PBS-T, and stored at 4°C. To allow for multiplexed assays, each antigen was coupled to distinct microspheres: GN on #19, NP on #22, GC on #30, and GP38 on #78.

Anti-CCHFV IgM or IgG prevalence was determined by single point dilution of sera using the multiplex CCHFV serology assay and read on the MAGPIX platform (Luminex) using 96-well plates. Sera was diluted 1:100 in 5% skim milk in PBS-T. Samples were incubated with microspheres for 1 h with 50 µL of sample and 2500 microspheres of each antigen per well. After incubation, microspheres in each well were washed three times with 100 µL of PBS-T. Goat α-mouse-IgG-PE was diluted 1:100 in 5% skim milk in PBS-T, applied to microspheres at 50 µL per well, and allowed to incubate for 1 h. Microspheres were washed three times with PBS-T, suspended in 100 µL of PBS-T, and read by the MAGPIX instrument. Samples were run in duplicate, and each plate included positive and negative control sera. Antibody positive samples were determined as those samples at which the signal was statistically different (µ + 3σ) from pre-bleed samples.

## 3 Results

### 3.1 Role of humoral immunity in vaccine-mediated protection against CCHFV

**Fig. 1:**
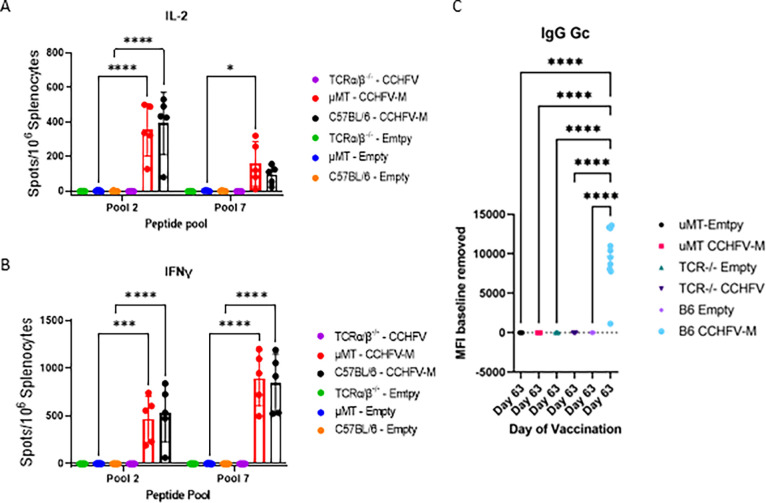
Immune response in CCHFV-MAfg-09 vaccinated T-cell and B-cell knockout IS mice. Groups of 15 mice were vaccinated three times with 50 µg of CCHFV-M_Afg-09_ or empty vector by IM-EP at three-week intervals. The splenocyte T-cell response of five mice per group was analyzed by ELISpot (B6 empty group n = 4, all other groups n = 5). Splenocytes from individual mice were restimulated with two pools of peptides derived from the CCHFV strain Afg09-2990 M-segment. Anti-CCHFV-M specific (A) IL-2+and (B) IFN-γ+ T cells were quantified by ELISpot. Data are the group mean averages ± SD. *p < 0.05; **p < 0.01; ***p < 0.001; ***p < 0.0001. P-values were determined by two-way ANOVA with Sidak's multiple comparison test with a 95% confidence interval. (C) anti-G_C_ antibody responses of vaccinated mice to be challenged was measured in the sera at three weeks after the final vaccination by Magpix.

To examine if the humoral and/or cellular immune responses are necessary for protection when mice were vaccinated with CCHFV-M_Afg09_, we took advantage of the different available KO mice. Mice lacking functional B cells (µMT^−/−^), functional α/β T cells (TCRα/β^−/−^) or C57BL/6 (B6) control mice were vaccinated with CCHFV-M_Afg09_ or the empty DNA vector. Fifteen mice per group were vaccinated three times at three-week intervals with 50 µg of DNA per vaccination by IM-EP. To verify if the µMT^−/−^ mice had comparable T-cell responses to the B6 controls, splenocyte T-cell responses (n = 4 or 5 mice per group) were measured against two previously described (Suschak et al., 2021) highly immunogenic regions of the GPC by ELISpot one week after the final vaccination (Fig. 1A and B). Peptide pool 2 spans the GP38 region, and peptide pool 7 spans the N-terminus of the G_C_. The interleukin-2 (IL-2) and interferon gamma (IFNγ) responses were not significantly different between the µMT^−/−^ and the B6 CCHFV-M_Afg09_ vaccinated groups with either peptide pool, suggesting the cellular immune response is similar between the two mouse strains and the absence of B cells is not significantly impacting the IL-2 and IFNγ T-cell responses. Consistent with absence of the TCRα/β T cells, no responses were detected in TCRα/β^−/−^ mice (Fig. 1A and B). Three weeks after the final vaccination, the humoral response was assessed to determine if the TCRα/β^−/−^ mice developed a similar antibody response to the B6 control mice. Specific anti-Gc IgM (Fig. S1) and IgG (Figs. 1C and S2) were measured using a multiplexed, magnetic bead based serologic assay developed for the MAGPIX system. IgM levels were significantly higher in B6 mice compared to TCRα/β^−/−^ mice when measured at day 63 post vaccination. IgM was absent from µMT^−/−^ mice. The B6 CCHFV-M_Afg09_ vaccinated group developed significantly higher levels of anti-G_C_ IgG compared with any of the other groups. No significant levels of IgM or IgG against both G_N_ and GP38 were detected (Figs. S1 and 2). These results suggest that the lack of CD4^+^ T-cell modulation prevented the isotype switch to IgG in the TCRα/β^−/−^ mice.

**Fig. 2:**
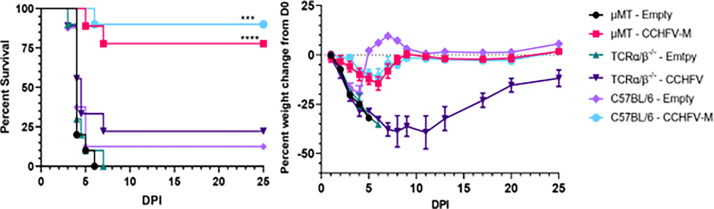
CCHFV challenge of vaccinated T- and B-cell deficient mice. Groups of 8-10 mice were challenged with 100 PFU of CCHFV strain Afg09-2990, µMT empty (n = 10), µMT CCHFV-M_Afg09_ (n = 9), TCRα/β^−/−^ empty (n = 10), TCRα/β^−/−^ CCHFV-M_Afg09_ (n = 9), B6 empty (n = 8), and B6 CCHFV-M_Afg09_ (n = 10). Survival and weight change from baseline on day 0. ***p = 0.0003 and ****P < 0.0001, comparison of CCHFV-M_Afg09_ to empty vector groups for each mouse species by log-rank test.

Because the IFN-I response in wild-type mice prevents CCHFV disease progression, four weeks following the final vaccination, mice (n = 8-10 per group) were treated with 2.0 mg of mAb-5A3 one day prior to challenge and an additional 0.5 mg dose was given again on day +1 to disrupt IFN-I activity (Garrison et al., 2017; Suschak et al., 2021). Mice were challenged with 100 PFU of CCHFV strain Afg09-2990 by the IP route. Mouse weight and survival were measured daily for 25 days post-infection (Fig. 2). The CCHFV-M_Afg09_ vaccinated µMT^−/−^ mice were significantly protected against lethal challenge with CCHFV Afg09-2990 (78% survival, 7/9) in comparison to the empty vector µMT^−/−^ controls (0/10 survived, *p* < 0.0001). In contrast, TCRα/β^−/−^ mice were not significantly protected against lethal disease (22% survival, 2/9). These findings suggest that the humoral responses are not essential for the protection observed with CCHFV M-segment targeting DNA vaccine. To the contrary, absence of TCRα/β T cells completely abrogate protection in this model. Considering the effects that the lack of CD4^+^ T cells can have on humoral responses and IgG class switching (Fig. 1C), we next addressed the specific contribution of CD8^+^ T cells.

### 3.2 Importance of CD8^+^ T cells on CCHFV DNA vaccine mediated protection

**Fig. 3:**
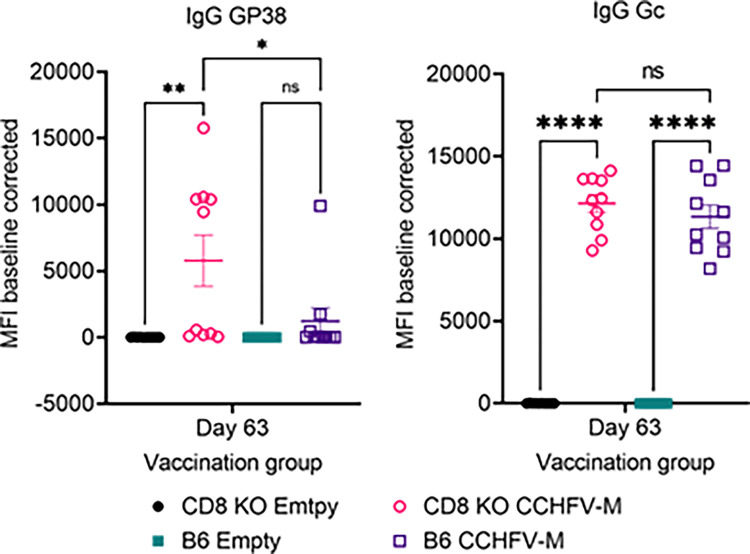
Humoral immune responses in CD8^−/−^ and B6 wild-type mice. The anti-GP38 and anti-G_C_ antibody responses of vaccinated mice to be challenged was measured in the sera at three weeks after final vaccination by MAGPIX.

The direct role for CD8^+^ T cell-mediated protection by the CCHFV-M_Afg09_ vaccine was assessed using CD8^−/−^ mice. Groups of 10 CD8^−/−^ mice and B6 controls were vaccinated with either the CCHFV-M_Afg09_ or the empty DNA vector, three vaccinations at three-week intervals with 50 µg per vaccination via IM-EM. Similar to wild-type C57BL/6 mice, CD8^−/−^ mice are not susceptible to lethal CCHFV infection when IFN-I activity is not blocked (Fig. S5). Humoral responses were assessed by MAGPIX binding assay in serum taken from vaccinated animals on day 63. All ten B6 and CD8^−/−^ mice had similar IgG responses against G_C_ (Fig. 3). CD8^−/−^ mice had higher IgG responses against GP38, another immuno-relevant vaccine and antibody target for CCHFV (Golden et al., 2019; Suschak et al., 2021). Only one CCHFV-M_Afg09_ vaccinated B6 mouse had a measurable response to G_N_ (Fig. S4). No responses were detected in the negative-control vaccinated animals. IgM response against GP38, G_N_, and G_C_ were comparatively lower than IgG responses for all groups (Fig. S3).

**Fig. 4:**
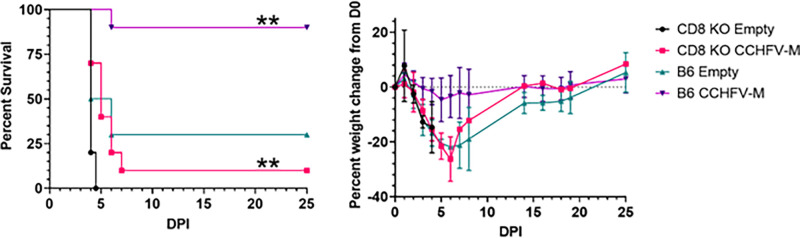
Survival and antibody response of CCHFV-M_Afg-09_ vaccinated CD8^+^ T-cells IS mice. Groups of 10 mice were vaccinated three times with 50 µg of CCHFV-M_Afg-09_ or empty vector by IM-EP at three-week intervals. Groups of 8–10 mice were challenged with 100 PFU of CCHFV strain Afg09-2990. Survival and weight change from baseline on day 0. **p < 0.01, comparison of CCHFV-M_Afg09_ to empty vector groups for each mouse species by log-rank test.

Four weeks following the final vaccination, mice were challenged with 100 PFU of CCHFV strain Afg09-2990 IP and monitored for 25 days. IFN-I was blocked using the mAb-5A3 antibody as above. Following CCHFV challenge, only 1/10 CCHFV-M vaccinated CD8^−/−^ mice survived compared to 9/10 B6 CCHFV-M vaccinated animals. There was a significant delay in mean time-to-death of one day (p = 0.0014) in CCHFV-M vaccinated CD8^−/−^ mice in comparison to CD8^−/−^ mice vaccinated with the empty vector (0/10 survived) (Fig. 4). Considering there were no significant differences in the humoral response between CD8^−/−^ and B6 vaccinated mice, these data suggested that the CD8^+^ T cells were sufficient for protection facilitated by the CCHFV-M_Afg09_ vaccine.

## 4 Discussion

Several studies have shown that CCHFV vaccines produce both humoral and cellular immunity, and the vaccines protect against infection. However, no study has definitively identified whether humoral and/or cellular immunity are critical elements for M-segment based vaccine-mediated protection. A study by Dowall et al. (2016) suggested that a modified vaccinia Ankara-based CCHFV vaccine encoding the glycoproteins required both the humoral and adaptive response. In contrast, the majority of CCHFV vaccine studies in both mice and NHPs have consistently revealed that the antibody response to the glycoproteins in vaccines does not correlate with protection (Garrison et al., 2017; Hawman et al., 2021; Hawman et al., 2022; Kortekaas et al., 2015; Leventhal et al., 2022). For example, we have previously shown that the DNA vaccine induces potent neutralizing responses in two mouse models, even at a suboptimal dose of 25 µg/ml, but the neutralizing response did not correlate with protection as some of the animals with the highest neutralizing titers succumbed to infection and some with the lowest responses survived (Garrison et al., 2017). Thus, circumstantially, cellular immunity appeared to be the most critical component of immune protection after vaccination, but an experimentally confirmed role for cell mediated protection has not been established. Previously the IFN-I blockade infection model was used to establish an important role for cytoplasmic pathogenic sensing and TNF-α signaling during CCHFV-mediated pathogenesis (Golden et al., 2022). Here we exploited the IFN-I blockade model to explore the contribution of adaptive immune components important for CCHFV vaccine-mediated protection. For vaccine studies the ability to transiently impair IFN-I activity has added value by allowing adaptive immune responses to develop in an IFN-I intact environment. IFN-I is only blocked during the viral challenge. Accordingly, vaccine facilitated immune responses are not impacted by congenital ablation of IFN-I. Our findings revealed that humoral responses were not sufficient for protection by the M-segment DNA vaccine. Contrastingly, loss of the CD8^+^ T-cell immune compartment was severely detrimental to protection, despite humoral responses similar to wild-type control mice. We conclude that protection incurred by the M-segment based DNA vaccination requires cell-based immunity, but humoral responses are dispensable. However, one potential limitation of our study is the fundamental differences in immune responses between different in-bred mouse strains (Radaelli et al., 2018). It is known that generally BALB/c mice produce stronger humoral responses compared to C57BL/6 mice, so we cannot discard the possibility that lack of protective humoral responses is related with this intrinsic phenotype. We believe this is not the case as significant humoral responses are detected in the C57BL/6 mouse model used in our study, but they do not correlate with protection.

Previously, we have shown through ELISpot analysis that the CD8^+^ T-cell response against CCHFV-M_Afg09_ is limited to GP38, NS_M_, and G_C_ in the IS model (Suschak et al., 2021). GP38 (when present) and G_C_ T-cell responses are consistently observed with other glycoprotein-based vaccines, and these responses vary in magnitude depending on the mouse strain used (Appelberg et al., 2022; Buttigieg et al., 2014; Hinkula et al., 2017; Saunders et al., 2023). Collectively, the T-cell response to G_C_ is consistent across various vaccine platforms and may represent an important correlate of protection in mice. The T-cell response against GP38, in glycoprotein-based vaccines that include this target, appears to be more variable depending on the mouse model used and may be due to the timing of the ELISpot analysis postvaccination, even with the same vaccine platform (Buttigieg et al., 2014; Saunders et al., 2023).

Despite the dispensability of humoral responses for protection in our vaccine model, our group and others have found that monoclonal antibodies targeting CCHFV can protect adult mouse models (Durie et al., 2022; Fels et al., 2021; Golden et al., 2019; Mishra et al., 2022). In those studies, monoclonal antibodies targeting a specific glycoprotein, called GP38, were protective. Neutralizing antibodies targeting G_C_ are more variable in protection, and to date only one engineered biscistronic antibody afforded therapeutic efficacy, whereby two neutralizing antibodies were combined that target distinct sites on one of the 6 identified antigenic sites in G_C_. These findings show that extrinsically delivered antibodies can be protective. There was a significant increase in the mean time-to-death of the CD8^−/−^ mice vaccinated with CCHFV-M_Afg09_ in comparison to the empty vector vaccinated group, which suggests the antibody response may have provided some level of protection. This may indicate that humoral responses against the glycoproteins induced by vaccination do not produce antibody against key protective epitopes at levels sufficient to incur robust protection.

In summary, here we show that the CD8^+^ T-cell response is crucial for the protective efficacy of the M-segment based DNA vaccine to CCHFV. Additional studies to examine the immune response to each component across vaccine platforms is needed to determine if our findings for GPC hold true beyond DNA vaccination. The knowledge gained from these studies could be used to improve the design of the GPC component of CCHFV vaccines, as antigenic strategies to target CD8^+^ T cells differ from a global strategy for the immune system (Cosma and Eisenlohr, 2018; Freitag et al., 2021; Gasper et al., 2016; Retamal-Diaz et al., 2019). Our work using the IFN-I blockade model in mice lacking specific adaptive immune compartments provides a template to explore those questions.

## Disclaimer

Opinions, interpretations, conclusions, and recommendations are those of the authors and are not necessarily endorsed by the U.S. Army.

## CRediT authorship contribution statement

**Joseph W. Golden:** Conceptualization, Methodology, Data curation, Formal analysis, Writing – original draft. **Collin J. Fitzpatrick:** Methodology, Data curation. **John J. Suschak:** Conceptualization, Methodology, Data curation. **Tamara L. Clements:** Methodology, Data curation. **Keersten M. Ricks:** Conceptualization, Methodology, Data curation, Formal analysis, Writing – original draft. **Mariano Sanchez-Lockhart:** Conceptualization, Methodology, Data curation, Writing – original draft. **Aura R. Garrison:** Conceptualization, Methodology, Data curation, Formal analysis, Funding acquisition, Writing – original draft.

## Declaration of Competing Interest

The authors declare that they have no known competing financial interests or personal relationships that could have appeared to influence the work reported in this paper.

## Data Availability

Data will be made available on request.

## Appendix Supplementary materials

Image, application 1
