# Supplementary material for: Induced protection from a CCHFV-M DNA vaccine requires CD8+ T cells
Source: Virus Res. 2023 Jul 24;334:199173. doi: 10.1016/j.virusres.2023.199173 (PMC10388194; doi:10.1016/j.virusres.2023.199173)
Supplement: Supplementary file 1 [file mmc1.docx]

**Supplementary figures for**

**Induced protection from a CCHFV-M DNA vaccine requires CD8^+^ T cells**

Joseph W. Golden^1,*^, Collin J. Fitzpatrick^1^, John J. Suschak^1^, Tamara L. Clements^3^, Keersten M. Ricks^3^, Mariano Sanchez-Lockhart^2^ and Aura R. Garrison^1, *^

*^1^Virology Division, ^2^Center for Genome Sciences, Molecular Biology Division and ^3^Diagnostic Systems Division, United States Army Medical Research Institute of Infectious Diseases, Fort Detrick, MD 21702*

*Corresponding authors: aura.r.garrison.civ@health.mil and joseph.w.golden.civ@health.mil

**This PDF file includes:**

Figs. S1 to S5

**Supplemental Figures**


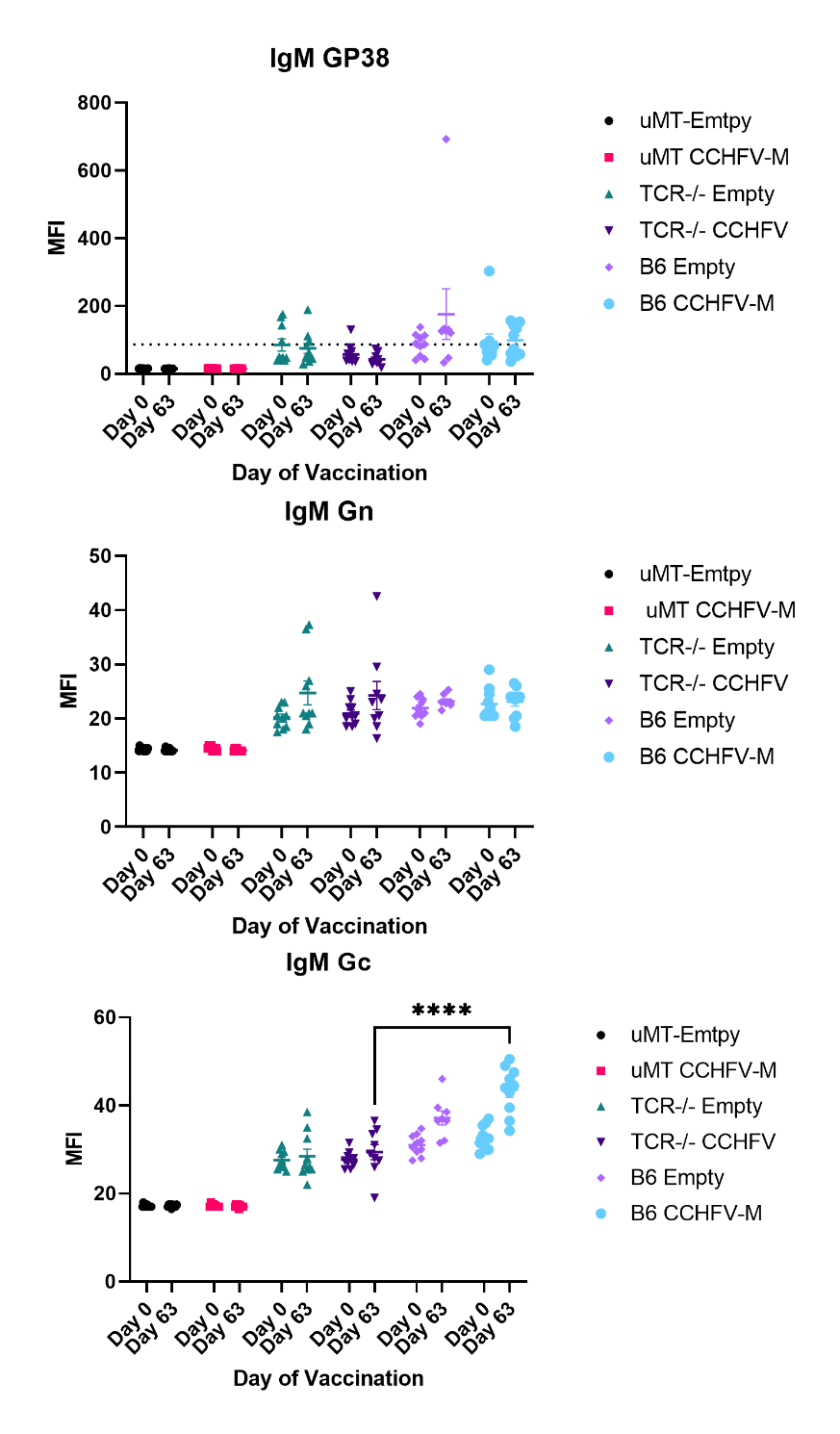


**Figure S1. Humoral immune responses in µMT, TCR^-/-^ and B6 wild-type mice.** The anti-GP38, anti-G_N_ and anti-G_C_ IgM antibody responses of vaccinated mice was measured in the sera at three weeks post-final vaccination by MAGPIX.


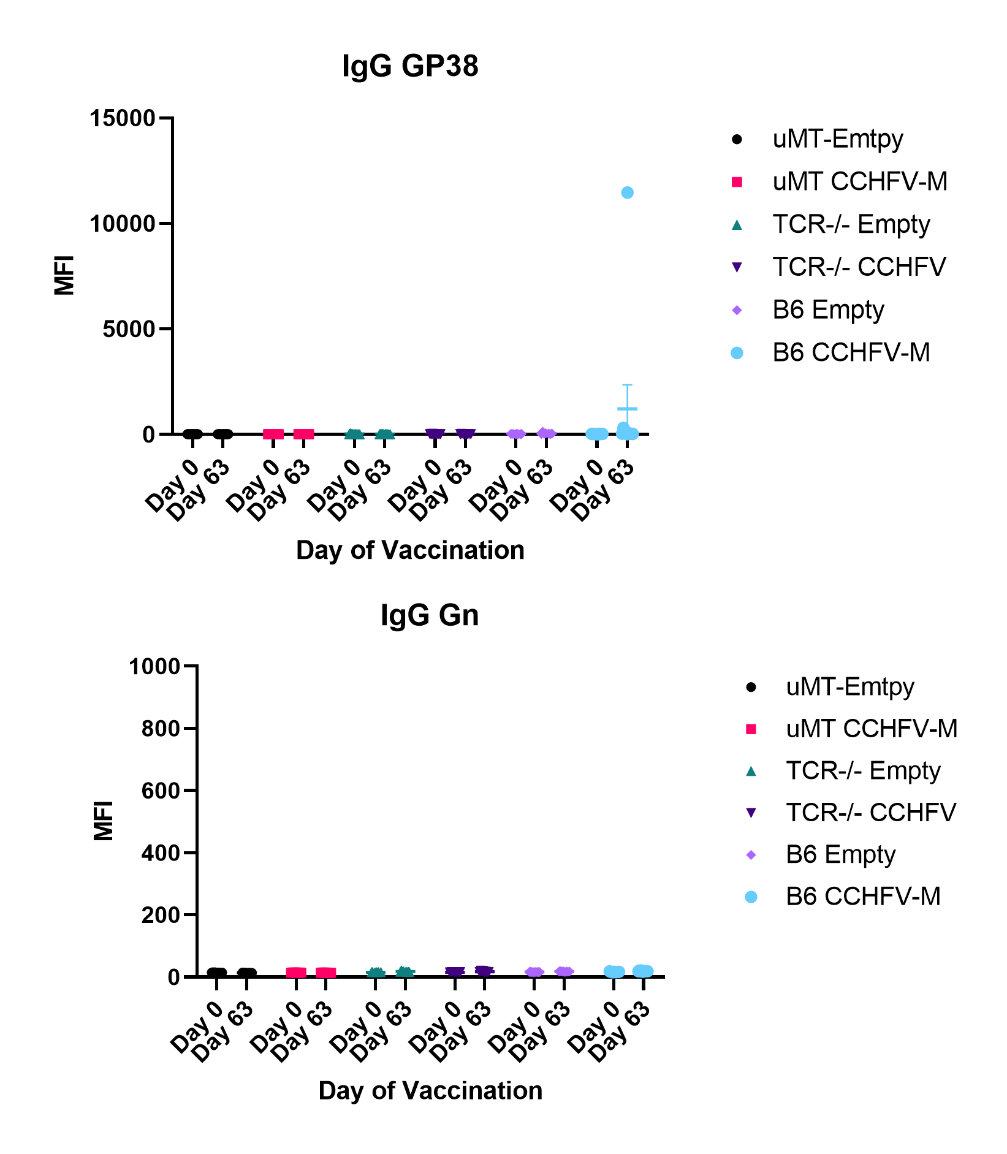


**Figure S2. Humoral immune responses in µMT, TCR^-/-^ and B6 wild-type mice.** The anti-GP38 and anti-G_N_ (IgG antibody responses of vaccinated mice was measured in the sera at three weeks post-final vaccination by MAGPIX.


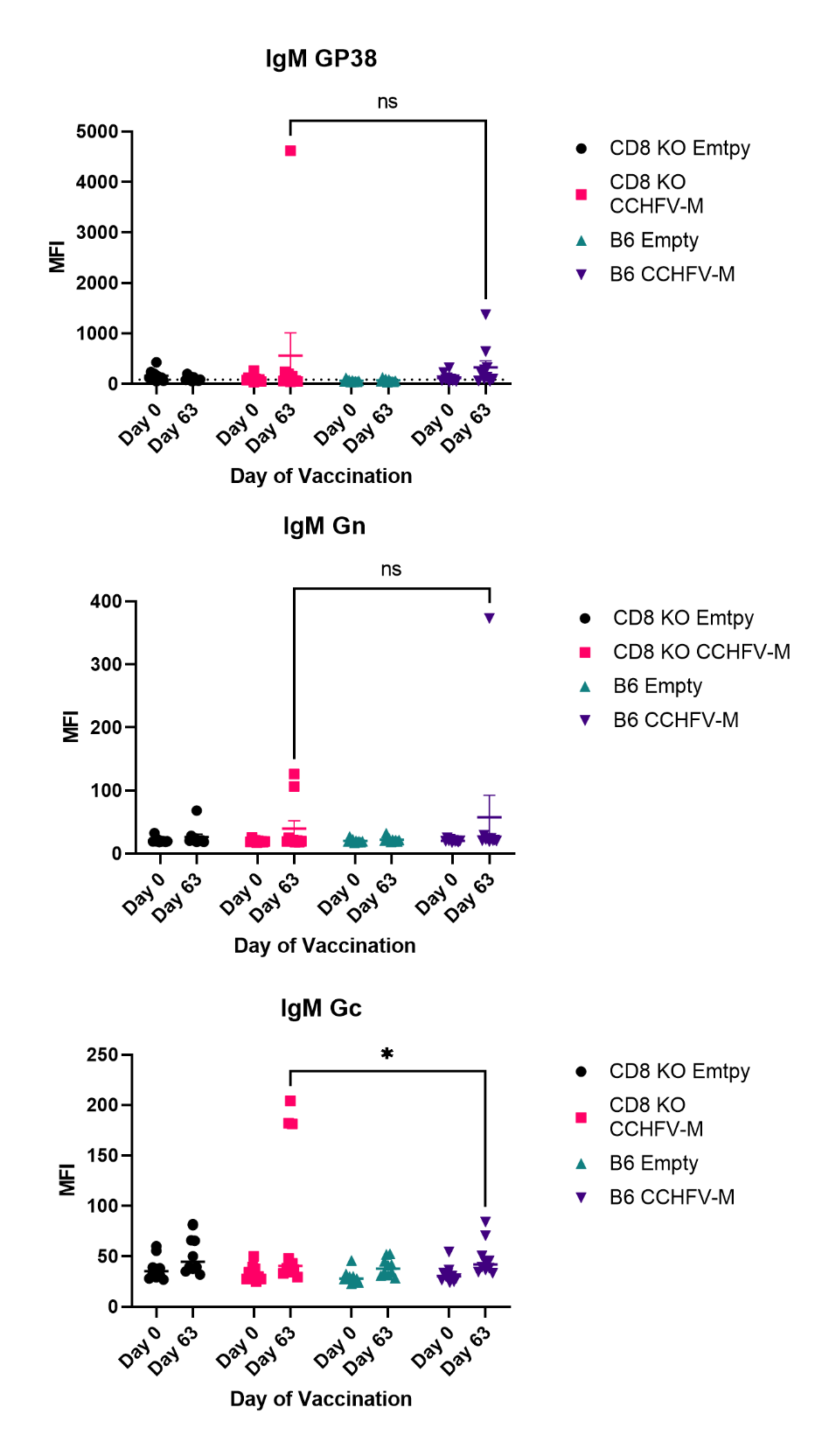


**Figure S3. Humoral immune responses in CD8^-/-^ and B6 wild-type mice.** The anti-GP38, anti-G_N_ and anti-G_C_ IgM antibody responses of vaccinated mice was measured in the sera at three weeks post-final vaccination by MAGPIX.

**Figure S4. Humoral immune responses in CD8^-/-^and B6 wild-type mice.** The anti-G_N_ IgG antibody responses of vaccinated mice was measured in the sera at three weeks post-final vaccination by MAGPIX.


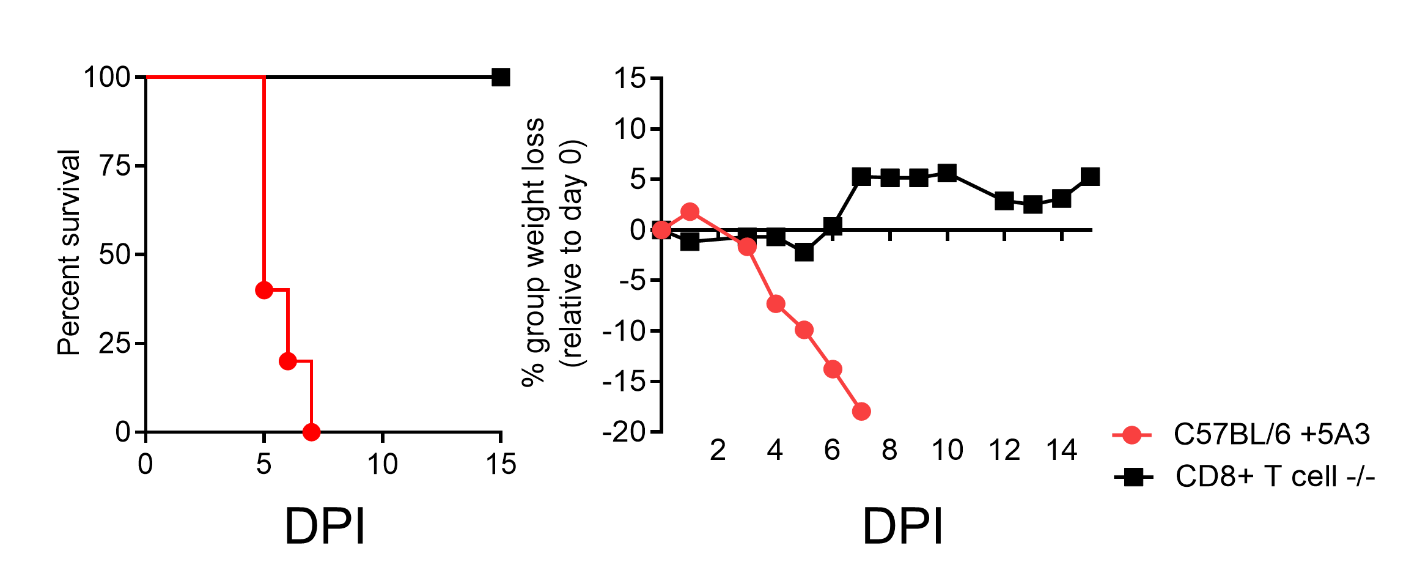


**Figure S5. CD8^+^ T-cell deficient mice are not susceptible to CCHFV infection when type I interferon is active.** C57BL/6 or CD8^+^ T-cell^-/-^ mice were infected with 100 PFU of CCHFV strain Afg09-2990 by the IP route. On day +1, C57BL/6 mice were treated with 2.5 mg of mAb-5A3 to block IFN-I activity. CD8 deficient mice were not treated. Survival (left) and weight change (right) from baseline on day 0.
